# A Novel-Potential Wave-Bump Yarn of Plain Weave Fabric for Fog Harvesting

**DOI:** 10.3390/molecules29214978

**Published:** 2024-10-22

**Authors:** Luc The Nguyen, Luu Hoang, Le Thuy Hang, Jiansheng Guo

**Affiliations:** 1 Faculty of Garment Technology and Fashion Design, Hung Yen University of Technology and Education, Hung Yen 160000, Vietnam; hanglt1983@gmail.com; 2 Key Laboratory of Textile Science and Technology, Ministry of Education, College of Textiles, Donghua University, Shanghai 201620, China; jsguo@dhu.edu.cn

**Keywords:** droplet capturing, droplet converging, droplet growing, droplet jumping, droplet shedding, lubricated, fabric, fog harvesting, PDMS, slippery, tetrabutyl titanate, wave-bump yarn

## Abstract

With the variety of fibers and fabrics, the studies of the surface structure of the textile yarns, the weave fabric, and their surface wettability are still potential factors to improve and optimize the fog harvesting efficiency. In this work, inspired by the fog harvesting behavior of the desert beetle dorsal surface, a wavy–bumpy structure of post-weave yarn (obtained from woven fabric) was reported to improve large droplet growth (converge) efficiency. In which, this study used tetrabutyl titanate (Ti(OC_4_H_9_)_4_) to waterproof, increase hydrophobicity, and stabilize the surface of yarns and fabric (inspired by the feather structure and lotus leaf surface). Moreover, PDMS oil was used (lubricated) to increase hydrophobicity and droplet shedding on the yarns (inspired by the slippery surface of the pitcher plant) and at the same time, enhance the fog harvesting efficiency of the warp yarn woven fabric (Warp@fabric). In addition, a three-dimensional adjacent yarn structure was arranged by two non-parallel fabric layers. The yarns of the inner and outer layers were intersected at an angle decreasing to zero (mimicking the water transport behavior of Shorebird’s beaks). This method helped large droplets quickly form and shed down easily. More than expected, the changes in fabric texture and fiber surface yielded an excellent result. The OBLWB-Warp@fabric’s water harvesting rate was about 700% higher than that of the original plain weave fabric (Original@fabric). OBLWB-Warp@fabric’s water harvesting rate was about 160% higher than that of Original–Warp@fabric. This shows the great practical application potential of woven fabrics with a low cost and large scale, or you can make use of textile wastes to collect fog, suitable for the current circular economy model. This study hopes to further enrich the materials used for fog harvesting.

## 1. Introduction

In modern society, people have been using groundwater for industrial production, agriculture, and services. Due to climate change, many areas have become barren and desertified. The problem of clean water is becoming more and more urgent not only in desert areas but also in other areas. Scientists have performed many studies to harvest clean water from nature. Methods include harvesting water from moisture in the air, from seawater, and from fog. However, to further improve the efficiency of water harvesting from fog, researchers around the globe are still actively researching, manufacturing, and modifying fog harvesting materials. These new materials and fog harvesters were inspired by the water harvesting abilities of many different species in nature [1,2,3,4,5]. One of the organisms was most interested in the special geometric structure of bumps and flexible wettable surface alternating hydrophobic/hydrophilic regions. That is the desert beetle [6,7,8]. These typical studies that mimic beetle behavior include research by Jun Kyu Park and Seok Kim (2019) [9], and they designed three-dimensional structure flexible fog harvesting surfaces inspired by Namib Desert beetles; Mallinath S. Birajdar and Jonghwi Lee fabricated nanoscale bumps and dents on nanofibers, enabling sonication-responsive wetting and improved moisture collection [10]; Lianbin Zhang et al. (2015) were inspired by the efficient fog harvesting behavior of Stenocara beetles in the Namib Desert. A mussel-inspired ink consisting of an optimized solution of dopamine was applied directly by inkjet printing to superhydrophobic surfaces [11].

Recently, a lot of new materials and fog harvesters have been created by scientists. Their studies not only mimicked the fog harvesting behavior of one species, but also integrated the superior properties of several different species. Inspired by the structures of the hairs of the trichomes of Sarracenia, a genus of carnivorous pitcher plants, a bionic high–low rib-like microstructure with superhydrophobic and directional water transport properties was reported by Jing Li et al. [12]. Pingan Zhu et al. drew their inspiration from the rugged shape of a Gunnera leaf to enhance fog deposition, the hierarchical surface roughness of a Cotula leaf to lubricate the pathway for rapid water drainage, and the heterogeneous wetting ability of the Namib Desert beetle to promote the directional water transport during fog deposition and water drainage [13]. Jian Wang et al. combined the advantages of cactus and Sarracenia to create a novel configuration, a spine with barbs and hierarchical channels (SBHCs), for simultaneous ultrafast water transport and highly efficient fog harvesting [14].

Among the materials and fog harvesters designed and fabricated, fabrics and textile materials have been gaining attention, typically the traditional polymer woven meshes [15,16,17]. Several studies for improving the weave and modification of fabric surfaces have also obtained remarkable achievements in using fabrics for fog harvesting. In the study of Yue Gao et al. (2018), they fabricated a weft-backed woven fabric with a hydrophilic–superhydrophobic pattern. Different proportions of viscose and PP yarns were designed to fabricate the hybrid surface. When the area was certain, it turned out that the prepared sample had the best water harvesting rate of 1267.5 mg h^−1^ cm^−2^ with a proportion of 1:1 (viscose yarn/PP yarn) [18]. The study by Zhihua Yu et al. (2020) contributed to a flexible and highly efficient fog collector that was prepared by mimicking the posterior exoskeleton structure of the Namib Desert beetle. The innovative fog collector was constructed by a superhydrophobic–superhydrophilic patterned fabric through a simple weaving method followed by the in situ deposition of copper particles. Compared with the conventional fog collector with a plane structure, the fabric has shown a higher water harvesting rate at 1432.7 mg/h/cm^2^ [19]. Ruofei Zhu et al. reported a facile method to prepare Janus fabrics with asymmetric wettability for on-demand oil/water separation and hydrophobic/hydrophilic patterned fabrics for efficient fog harvesting. The photocatalytic degradation properties of PDVB were utilized to prepare Janus fabric with asymmetric wettability. For the patterned fabric with larger hydrophobic/hydrophilic areas, the water collection rate reached 224.7 mg cm^−2^ h^−1^ [20]. The limitation of these studies is that they do not take into account the dynamic factors (shade coefficient, Stokes number, etc.) affecting the water harvesting performance of the fabric in realistic fog and wind. Additionally, a yarn surface that had slippery wave-bumps to promote droplet growth and droplet shedding was also not considered [21,22,23,24].

In our previous studies, bumps on the filament surface have effectively supported droplet capture and droplet growth [25,26,27]. However, the creation of these bumps required multiple steps, both physical and chemical. In this study, the creation of bumps was simpler and more economical. A comprehensive solution for greening and material recycling has been effectively utilized in fog harvesting. Specifically, the plain weave fabric structure was modified to improve the fog harvesting efficiency based on dynamic factors affecting the water harvesting efficiency of the fabric. In particular, the post-woven yarn structure of woven fabrics was exploited and modified effectively. This yarn structure is inspired by the integration of beetles and other organisms to optimize water harvesting rates. Firstly, the wavy structure of the yarns consists of two interlaced regions, a concave and a convex region (similar to a beetle dorsal presentation). Secondly, the yarn surface was composed of many micro-fibers combined with Titanium nanoparticles, which are the product of the Sol–Gel tetrabutyl titanate (Ti(OC_4_H_9_)_4_) process [28,29,30]. This structure was inspired by the surface of bird feathers and the lotus leaves [31,32,33]. This increased the hydrophobicity of the yarn surface. Thirdly, the yarns are arranged spatially by joining two layers of Warp@fabric together, which are erected and intersected with a decreasing angle to zero. The arrangement of two layers of fabric did not parallel to each other but formed an angle; this innovative design was inspired by the ability of Shorebird’s beaks to transport and converge droplets. This aided in promoting superior large droplet growth (creating a water bridge) [34,35,36,37,38,39,40]. The fourth was inspired by the slippery surface of the pitcher plant; the yarn surface was impregnated—coated by PDMS oil (Polydimethylsiloxane oil) as a surface lubricant to increase droplet shedding/slipping potential [41,42,43,44,45,46]. These designs have helped the droplet behavior on the 3D fiber system for fog harvesting to become more optimal, efficient droplet capture, flexible droplet jumping, fast droplet sliding, and droplet converging with a very large volume.

## 2. Experiment

### 2.1. Preparation of Samples

**Figure 1:**
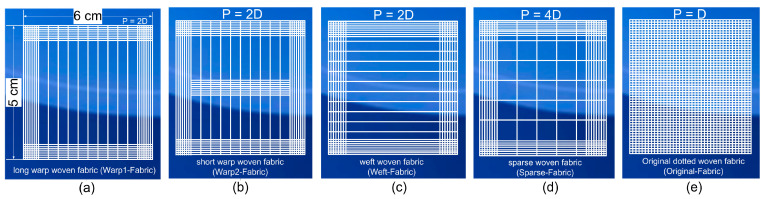
The preparation of the original woven fabric sample and the weave-modified fabric samples by varying the warp and weft ratio. (**a**) Warp@fabric. (**b**) Warp1@fabric. (**c**) Weft@fabric. (**d**) Sparse@fabric. (**e**) Original@fabric.

PET-Plain Weave Fabric was purchased from a local store. The width of each yarn is 0.38 mm. Each yarn consists of micro-fibers with a diameter of 30 µm. Fabric samples were prepared with dimensions D × R = 5 × 6 cm. Then, fabrics were modified by changing the ratio between the number of weft and warp yarns, corresponding to the other 5 samples as shown in Figure 1.

**Figure 2:**
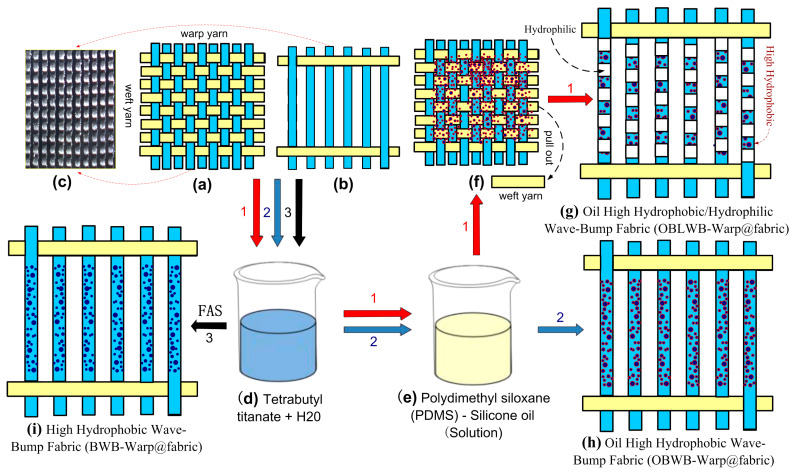
Illustrated procedure for preparing modified warp fabric samples. (**a**) Original@fabric. (**b**) Warp@fabric. (**c**) Actual sample of Warp@fabric. (**d**) Mixed solution after Sol–Gel tetrabutyl titanate process (mixture solution A). (**e**) PDMS oil. (**f**) OBWB@fabric. (**g**) OBLWB-Warp@fabric. (**h**) OBWB-Warp@fabric. (**i**) BWB-Warp@fabric.

The original Warp@fabric (Warp@fabric) surface was modified to create three different fabrics as shown in Figure 2 (corresponding to three different yarns). The detailed process was performed as follows (Figure 2). For sample 1, we used 15 g of tetrabutyl titanate (Ti(OC_4_H_9_)_4_) purchased from Shanghai Aladdin Bio-Chem Technology Co., Ltd., Shanghai, China; 1 g of H_2_O; and 0.05 g of NH_3_ and magnetically stirred for 4 h, and waterproofing compound A was fabricated. Subsequently, the fabric samples were impregnated in the A mixture solution. Then, the fabric surface was rubbed so that the TiO_2_ nanoparticle could penetrate well into the fibers of the yarns (Step 1). We continued to soak the fabrics in a (3,3,3-Trifluoropropyl) methydichlorosilane (FAS) solution for a few more hours (Step 2) [30]. Then, the High-Hydrophobic Wave-Bump Fabric sample (BWB-Warp@fabric) was obtained. For samples 2 and 3, the first step (Step 1) is the same as Step 1 of sample 1. Then, we added a certain amount of PDMS oil purchased from Xilong Science Co., Ltd., Guangzhou, China, stirred well, and continued rubbing and impregnating for several hours (Step 2). Finally, two modified surface fabric samples were obtained: Oil High-Hydrophobic Wave-Bump Warp@fabric (OBWB-Warp@fabric) and Oil High-Hydrophobic/Hydrophilic Wave-Bump Warp@fabric (OBLWB-Warp@fabric).

### 2.2. Characterization

**Figure 3:**
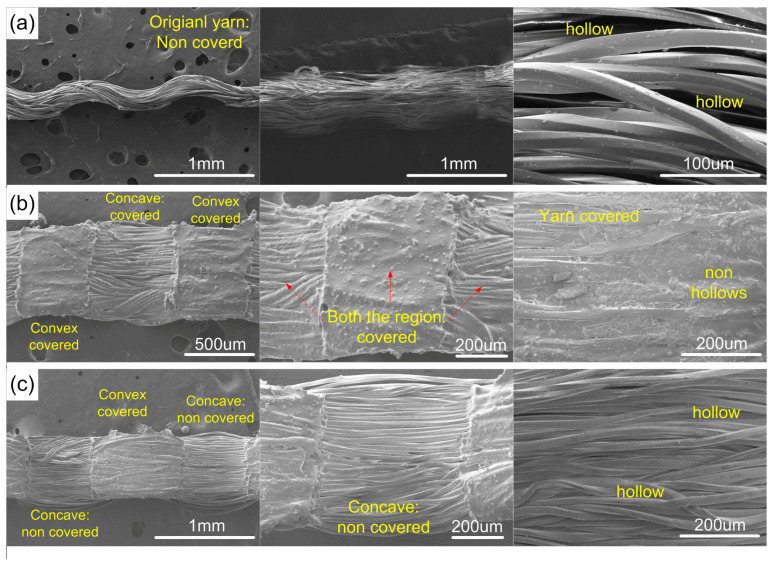
SEM images of original and modified yarns: (**a**) original yarn, LWB yarn, BWB yarn; (**b**) OBWB yarn; (**c**) OBLWB yarn.

**Figure 4:**
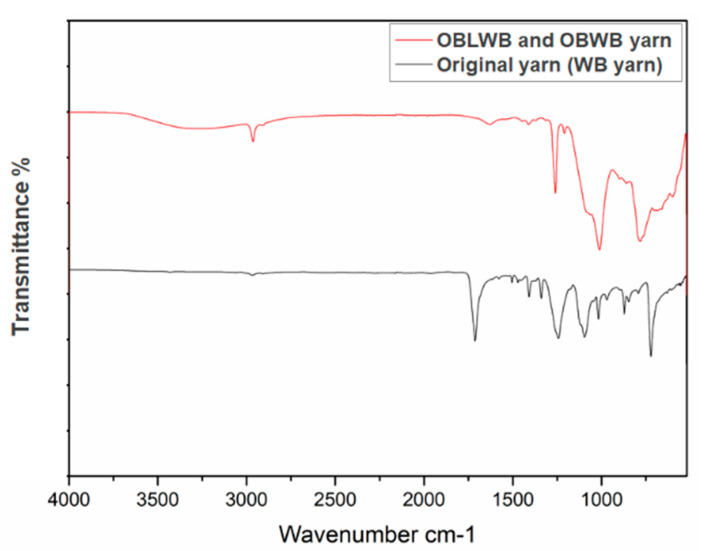
FTIR spectra of PET fabrics and PET filaments before and after modification: original PET yarn/OBW yarn and OBLW yarn.

Morphology of the fabric and yarn surface structure were observed by the JSM-7600F Schottky Field Emission Scanning Electron Microscope. A chemical analysis was achieved by The Nicolet iS10 FTIR Spectrometer (Figure 3 and Figure 4). The droplet behavior on the yarn surface was recorded by a Panasonic HC-X920M. The contact angle was calculated by a computer, camera, and software. The installed test chamber has the following dimensions: 200 × 200 × 200 mm. The temperature of 23 °C was controlled by the air conditioner. Humidity at 90% was maintained. The fog harvesting rate is performed by the fog generator [39].

## 3. Results

### 3.1. Surface Wettability of Modified Fabrics

**Figure 5:**
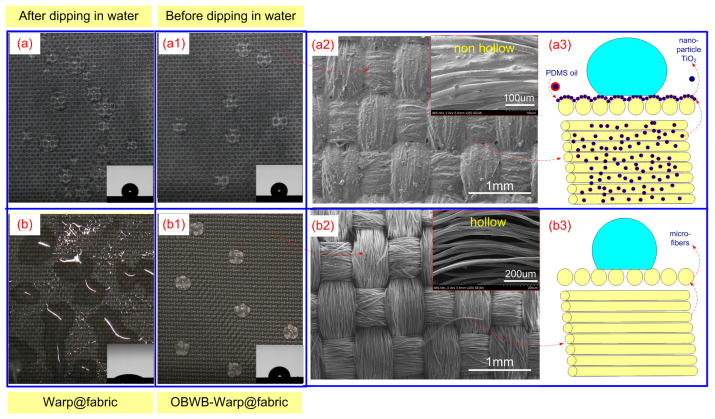
(**a**), (**a1**) The droplet shape and water contact angle on the surface of OBWB@fabric (before water immersion and after immersion, respectively); (**a2**) the SEM image of the OBWB@fabric surface; (**a3**) the illustration of the surface structure of OBWB@fabric. (**b**), (**b1**) The droplet shape and water contact angle on the surface of Original@fabric (before immersion and after immersion, respectively); (**b2**) the SEM image of the Original@fabric surface and surface structure of feathers; (**b3**) the illustration of the surface texture of Original@fabric.

Figure 5(b2,b3) show that the structure of Original@fabric is almost similar to that of a feather consisting of parallel fibers [31,32]. In which, each yarn consists of micro-fibers with a diameter of 30 µm. According to Wenzel’s theoretical model (Equation (1)), the surface of PET Original@fabric will become more hydrophobic than the smooth surface of PET (WCA = 90.2 °C).

(1) $$\cos\theta^{\prime }=rcos\theta$$

The Original@fabric’s water contact angle measured is 103.6 °C (Figure 5(b2)). However, if this fabric is used to harvest fog, the water will interact continuously with the fabric surface. After a while, water will penetrate deep into the yarn, because there are gaps between the fibers inside the yarn (Figure 5(b3)). This results in the fabric becoming more hydrophilic. Figure 5(b1) shows that the contact angle of Original@fabric after being immersed in water (LBW@fabric) is 25.8 °C.

To increase hydrophobicity and stability of the fabric surface during fog harvesting, a fabric surface modification solution was implemented according to the procedure in Figure 2. Figure 5(a1) shows that the WCA of OBWB@fabric increased to 122.7 °C. Furthermore, surface wetting stability was maintained and ensured through the immersion of OBWB@fabric in water. Then, after its contact angle, in which the WCA was changed, it is still 122.7 °C. The excellent combination of TiO_2_ particles in an A mixture solution with micro-fibers made the OBWB@fabric surface highly hydrophobic. The nanoparticle size, together with the viscoelastic support of the A mixture solution and the mechanical rubbing, has led the nanoparticles to penetrate deeply into the fabric structure. TiO_2_ nanoparticles adhered to the yarns and the gaps between the fibers in the yarn. This made the surface and internal structure of the yarn tight, non-hollow (Figure 3 and Figure 5(b3)), and stable. The increase in the contact angle and hydrophobicity can be transitioned from the Welzel model (Equation (1)) to the Cassie and Baxter state (Equation (2)). When a water droplet sits on the surface of OBWB@fabric, its wetting behavior can be described by the equation of Cassie and Baxter [29,47]:

(2) $$\cos\theta_{CB}=f_{ls}\cos\theta_{0}-f_{lv}$$

where *θ_CB_* is the WCA observed on a rough and porous surface, *θ*_0_ is the intrinsic WCA on the corresponding smooth surface, *f_ls_* is the liquid/solid contact area divided by the projected area, and *f_lv_* is the liquid/vapor contact area divided by the projected area.

On the other hand, part of the surface chemistry of the pristine PET fabric may have been altered by the infiltration and interaction of tetrabutyl titanate, H_2_O, NH_3_, FAS and PDMS. This can be shown by changing the FTIR spectral lines before and after modifying the PET fabric (Figure 4 FTIR image).

**Figure 6:**
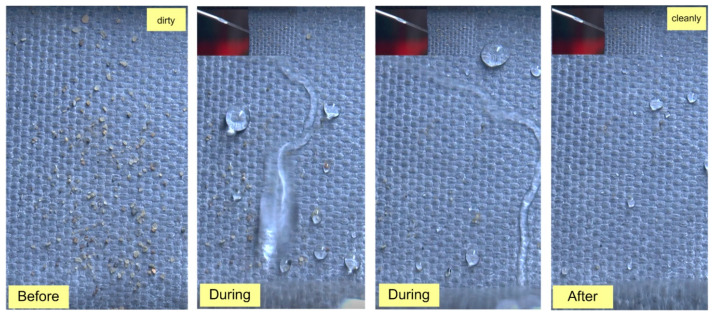
Hydrophobic droplet morphology and self-cleaning characterization on OBWB@fabric surface.

Moreover, the hydrophobicity and surface self-cleaning ability of OBWB@fabric are also recorded in Figure 6.

### 3.2. Aerodynamics Factor and Water Harvesting Efficiency of Modified Fabrics

**Figure 7:**
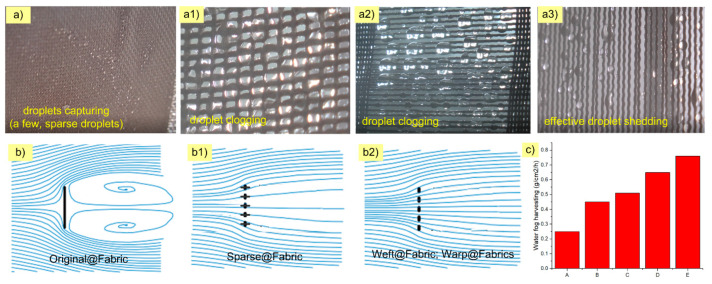
(**a**,**a1**,**a2**,**a3**) Droplet morphology on Original@fabric, Sparse@fabric, Weft@fabric, and Warp@fabric fabric surfaces, respectively. (**b**,**b1**,**b2**) The description of the fog stream that interacts with the fabrics Original@fabric, Sparse@fabric, Weft@fabric, and Warp@fabric, respectively. (**c**) The water harvesting rate of the fabrics: Original@fabric (A), Sparse@fabric (B), Weft@fabric (C), Warp1@fabric (D), and Warp@fabric (E).

According to previous studies on traditional fog harvesting mesh, the mesh cannot collect all the drops in the fog stream. Only a part of the droplet impacts the fiber surface and moves down the water harvester. The remainder of the fog does not impact the fiber surface. The first reason is that some flows through the holes of the mesh. The number of holes in the mesh affects the amount of mist impacting the yarn. The second one is due to bouncing back into the airstream. The water harvester texture or the mesh structure prevents the fog flow; a part of the fog flow will change and move around the mesh. If the mesh weave is too sparse, the amount of fog impacting the fiber surface is small, and the fog flow is lost a lot through the mesh. If the yarn is too tight–dense, the mesh itself will create resistance, causing the fog flow to change direction beyond the mesh edge; the amount of fog impacting the fiber surface will also be reduced (Figure 7b).

Additionally, the water collection capacity of a fog harvester depends on many factors such as wind speed, the liquid water content in fog, droplet size distribution, mesh characteristics, fiber diameter, etc. Briefly, there are two factors related to the water harvesting efficiency of fog harvesters: the shade coefficient and fiber characteristics. In this study, before considering yarn surface modification factors to improve the water harvesting rate, it is necessary to select the type of fabric corresponding to different weaves with the best shade coefficient to enhance water harvesting efficiency. Under the same conditions, the shading coefficient is one of the main factors affecting the water harvesting efficiency of the five modified fabrics (Original@fabric, Sparse@fabric, Weft@fabric, Warp@fabric, and Warp1@fabric). According to research by Juan de Dios Rivera [21] the shade coefficient represents the part of the harvester’s area capable of capturing droplets. It is represented by two equations:

*SC* = 1 − *f* (2); and *f* = *A*′/*A* (3)

where *f* is the free flow area ratio, *A*′ is defined as the mesh hole area, and *A* is the total screen area. According to Equations (2) and (3), and Figure 7(b,b1,b2), it can be partly explained regarding the factors leading to the difference in water harvesting rates of the five fabrics. Specifically, Figure 7c shows that the water harvesting rate of Original@fabric is the lowest (0.25 g/cm^2^/h). The reason is that the warp and weft density of the spot weave is very tight and the gap between the yarns is very small. The fog stream is redirected and moves around the fabric. The percentage of fog flow current passing through the fabric is very low (Figure 7b). According to Equations (2) and (3), the value of Original@fabric is very small, almost 0; the *SC* value is very large, almost 1. However, when analyzing comprehensively and completely, in addition to the shade coefficient affecting the water harvesting efficiency, according to the studies by Juan de Dios Rivera (2011) [21], Kyoo-Chul Park et al. (2013) [22], and Weiwei Shi et al. (2018) [24], they indicated that fog harvesting efficiency also depends on “pressure drop: *C*_0_” (also called “aerodynamic collection efficiency”). It represents the portion of droplets in the unperturbed fog that would collide with the fabric. In other words, the water harvesting rate directly correlates with a structure’s overall fog harvester (fabric) efficiency:

(4) $$\eta =\eta_{a}\eta_{d}$$

(5) $$\eta_{a}=\frac{SC}{1+{\left(C_{0}/C_{d}\right)}^{1/2}}$$

(6) $$\eta_{d}=\frac{SC}{S_{t}+\pi /2}$$

(7) $$S_{t}=\frac{2\rho_{\mathrm{water}}v_{0}{r_{\mathrm{fog}}}^{2}}{9\mu_{\mathrm{air}}R_{\mathrm{yarn}}}$$

where *η_a_* is the aerodynamic efficiency of the wind stream; *η_d_* is the deposition efficiency of fog droplets suspended in the wind passing through the yarns; *C*_0_ is the pressure drop coefficient of the fabric and *C_d_* is the drag coefficient for an equivalently shaped plate that is impermeable; ρwater is the density of water; and Ryarn is the yarn radius (width of yarn). Therefore, according to Equations (4) and (5), the fog harvesting efficiency depends on *C*_0_. Note that the drag coefficient *C_d_* in this equation corresponds to the impermeable screen; therefore, it is independent of the shade coefficient (*SC*). However, the pressure loss coefficient *C*_0_ depends on the *SC* value of Equation (8), where *k_Re_* is an empirical correction factor [21]:

(8) C0=kRe1.3SC+SC1−SC2

This implies that if *C*_0_ is too large, *ηa* will decrease. In the case of Original@fabric, the pressure drop coefficient (*C*_0_) was too large, leading to the original fabric fog harvest rate being very low. Furthermore, *η_a_* is related to the drag of the yarn structure with the reduction in the wind velocity. By the conservation of mass, the cross-sectional area of the upstream wind will pass through the fog structure continually with increasing drag, which will diminish the amount of heading towards the harvester. When the resistance makes the fog stream unable to pass through the yarns, it also means that the Stokes number (*S_t_*) decreases and the fog deposition efficiency *η_d_* decreases. Equation (4) and previous studies have shown that to improve the fog harvesting efficiency, the relationship between the shadow coefficient (*SC*) and Stokes number (*S_t_*) is quite complicated. Therefore, the selection of technical parameters relating to weaving of the fabric and yarn characteristics is very important.

Sparce@fabric’s water harvesting rate was significantly improved when the number of warp and weft yarns was removed (0.45 g/cm^2^/h). At this time, the characteristic shape of the fabric was almost similar to the traditional mesh. The distance between adjacent warp and weft threads was *p* = 4D (center to center). The shading coefficient of Sparce@fabric was lower than that of Original@fabric. However, the pressure drop coefficient (*C*_0_) of the Sparce@fabric was lower than that of Original@fabric. Furthermore, the fog droplet deposition efficiency and Stokes number *(S_t_*) increased due to more impactful fog streams and more deposition of fog droplets into the water reservoir. Therefore, the fog harvesting efficiency of Sparce@fabric was higher than that of Original@fabric.

Weft@fabric has *SC* roughly equivalent to that of Sparce@fabric. However, the water harvesting rate of Weft@fabric (0.51 g/cm^2^/h) was higher than that of Sparce@fabric. This is because the weave of the fabric has affected their fog harvesting efficiency. Previous studies by Kyoo-Chul Park et al. and Weiwei Shi et al. have indicated that the intersection between warp yarn and weft yarn leads to droplet clogging, while preventing flow and fog deposition efficiency (*S_t_*). This is one of the main reasons why Weft@fabric’s water harvest rate is lower than that of Sparce@fabric. However, the water harvesting rate of Weft@fabric is lower than that of Warp@fabric. Both Weft@fabric and Warp@fabric have the same *SC*, but the critical droplet volume on Weft@fabric (7 μL) is larger than that of Warp@fabric (6 μL). This leads to the droplet shedding efficiency of Weft@fabric being lower than that of Warp@fabric. In other words, on Weft@fabric, there is still a certain amount of droplet clogging.

As expected, the water harvesting rates of the Warp@fabric and Warp1@fabric warp samples were 0.65 g/cm^2^/h and 0.76 g/cm^2^/h, respectively, outperforming the above three (Figure 7c). This result is similar to the previous study on “Fog Harvesting with Harps” by Weiwei Shi et al. as well as our previous study on the “Vertical Filament Mesh” [25]. Under the effect of the force of gravity, the droplet on the warp yarn has the better shedding droplet ability than yarns with other geometric directions. The above theoretical equations of the authors Juan de Dios Rivera, Kyoo-Chul Park et al., and Weiwei Shi et al. have also shown that the Warp@fabric integrated the suitable shadow coefficient (SC: *p* = 2D) and high fog deposition efficiency—Stokes number (*S_t_*). In which, the outstanding advantage is the ability of droplet capturing and droplet shedding.

### 3.3. Droplet Behavior on the Surface of Wave-Bump Yarn and the Water Harvest Rate of Warp@Fabric

**Figure 8:**
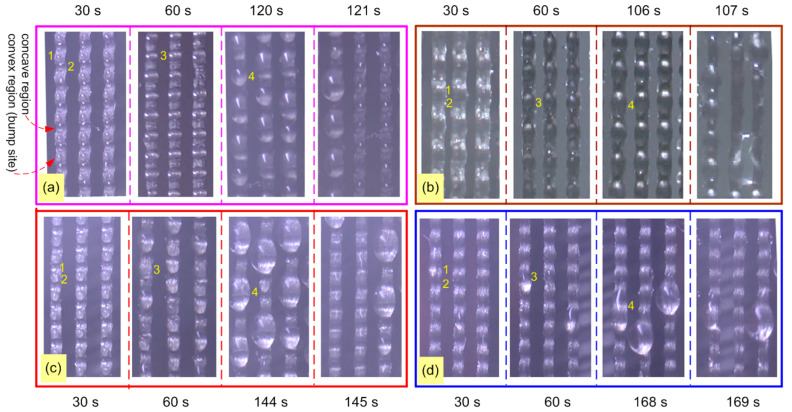
Droplet formation process—droplet behavior on the surface of 4 wave-bump yarns/fabrics at different times: (**a**) OBWB yarn/OBWB-Warp@fabric, (**b**) OBLWB yarn/OBLWB-Warp@fabric, (**c**) BWB yarn/BWB-Warp@fabric, (**d**) LBW yarn/Original–Warp@fabric.

**Figure 9:**
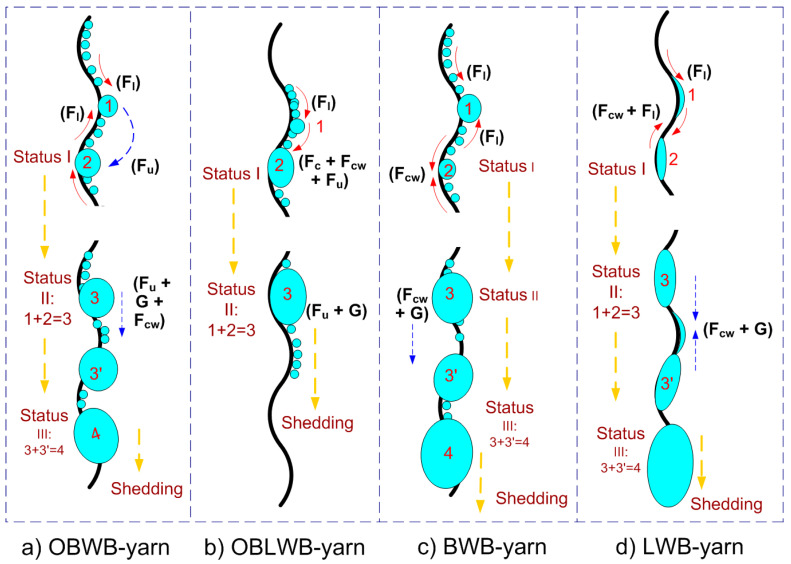
The illustration of droplet behavior on the surface of 4 fibers: (**a**) OBWB yarn, (**b**) OBLWB yarn, (**c**) BWB yarn and (**d**) LBW yarn. (*F_l_*) is the driving force generated by the shape gradient that propels a liquid drop towards the region with a larger curvature radius. (*F_c_*) is the driving force generated by the surface wettability gradient that propels liquid droplets towards the wetter region. (*F_wc_*) is the driving force generated by the surface wettability gradient that propels liquid droplets towards the wetter region of water. (*F_u_*) is the driving force generated by a surface lubricant. (*G*) is the force of gravity.

Figure 8 and Figure 9 show the droplet behavior on four types of wave-bump yarn (OBWB yarn, OBLWB yarn, BWB yarn, and LWB yarn). Initially, tiny droplets on the yarn surface rapidly formed; droplets transported and converged to the concave surface (wave bottom) of the yarn or the convex surface (bumpy wave top). However, the droplet behavior, droplet state, droplet transport time, convergence mode, and droplet shedding efficiency are different. That process is analyzed in detail as follows:

The first thing is the droplet behavior on the BWB yarn. The entire fiber surface is highly hydrophobic with a contact angle of 114.5 °C. Large drops quickly formed on both convex and concave regions. However, the convex region converged droplets that are larger than that of the concave region in the same unit of time. Then, larger droplets were formed in the entire convex region and part of the concave region. More specifically, initially, small round droplets were formed at both the bump and wave bottom of the yarn. At the wave bottom, the round droplet was immediately trapped and formed a more hydrophilic region (WR) by the hydrophilic properties of the –OH group in the water. At this time, the force Fwc appeared to push the droplets adjacent to the wave bottom towards the WR. From this, larger droplets formed. In contrast, in the bump region, initially, the small round droplets have adhered to the wave top. Because of the spherical shape of the bump, Flaplace (*F_l_*) propelled water droplets towards the region with a larger curvature radius. Then, two round droplets at the top and bottom of the wave keep getting bigger and bigger until their size is larger than the distance from the wave top to wave bottom (Figure 9c); they will converge to form a larger droplet. The final stage is that they reach the critical volume (5 μL) and slide down at the time (145 s).

Secondly, for the OBWB yarn, the entire fiber surface is not only highly hydrophobic (WCA = 122.7 °C) but also has the slippery properties of PDMS oil. Large drops quickly formed in both convex and concave regions. However, the droplet in the concave region was larger than that in the convex area in the same time unit. Due to the slippery surface, the droplet in the convex region quickly shed into the concave region, which lies neatly in this region. Then, larger droplets formed. The advantage of the corrugated structure helps to quickly form large droplets. Moreover, owing to the lubricated surface, the large droplet also quickly shed down at 121 s, which is earlier than that of the BWB yarn. The critical volume of the OBWB yarn fabric was smaller than that of the BWB yarn: 4.5 μL (Figure 8a and Figure 9a).

Thirdly, for OBLWB yarn, in the first place, the droplet on the convex surface is converged by Laplace force. Simultaneously, large droplets are formed very quickly in the concave region due to the spread of droplets on the hydrophilic surface (WCA = 25.8 °C). Then, under the effect of F_chem_ (*F_c_*) and *F_wc_*, the droplets in the convex region quickly moved to the more hydrophilic concave region. Large droplets were formed in a part of the convex region and the whole concave region. In this case, the dominant advantage is the extremely rapid formation of large droplets as the hydrophilic region interspersed the highly hydrophobic one, the lubricated surface, and the droplet propagation. What is more, PDMS’s lubricating oil and gravity made it easier for large droplets to shed down than in other cases. The large droplets on the yarn surface are quite long and close to each other, so it is also easy for them to attract each other to slide down faster. Droplets shed down quite early compared to the other cases (107 s). The critical droplet volume was 4 μL (Figure 8b and Figure 9b).

Fourthly, for the original yarn sample, the droplet formation process is divided into two stages. In stage 1, the droplet behavior on the original yarn is similar to that of the BWB yarn because the original yarn surface is also highly hydrophobic at first (WCA = 103.6 °C). After some time, the entire yarn was wetted as water had penetrated into the grooves between the fibers and the gaps between these fibers (Figure 3 and Figure 5(b2)). At this time, the original yarn surface became very hydrophilic and was called “LWB yarn”; the droplet behavior has been changed to phase 2. In this phase 2, after the fog had impacted the LWB yarn surface, a water film was formed in both the convex and concave regions of the yarn. Then, only a few large droplets were formed. The critical volume was 5.5 μL (Figure 8d and Figure 9d). It took quite a long time for the large droplet to shed (169 s).

**Figure 10:**
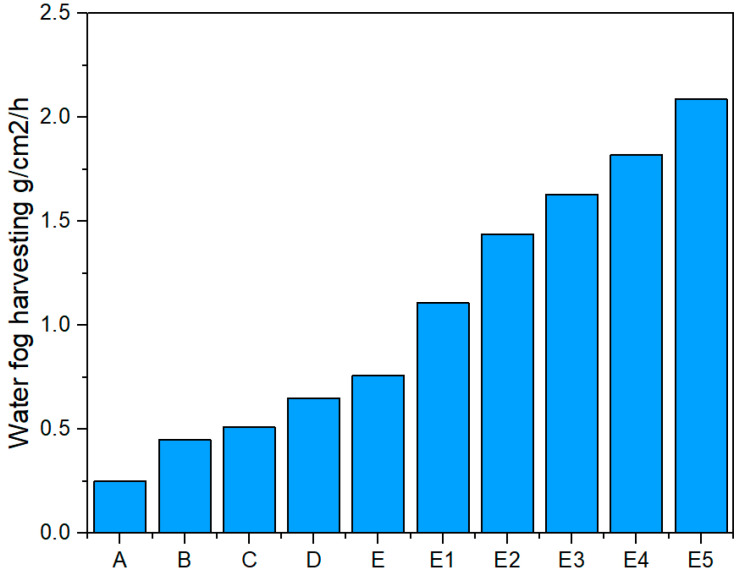
Water harvest rates of all modified fabric and original fabric samples. Meanwhile, A, B, C, D, E, E1, E2, E3, E4, and E5 are Original@fabric, Sparse@fabric, Weft@fabric, Warp@fabric (Original–Warp@fabric), BWB-Warp@fabric, OBWB-Warp@fabric, OBLWB-Warp@fabric, double-layer OBLWB-Warp@fabric (parallel), and double-layer OBLWB-Warp@fabric (non-parallel), respectively.

Figure 10 shows that the results obtained on the water harvesting rate of the fabric samples corresponding to the yarn types are also consistent with the above analysis. Specifically, the water harvesting rate of oil-lubricated fabrics (OBLBW-Warp@fabric and OBBW-Warp@fabric) is higher than that of other fabrics. Among them, the water harvesting rate of OBLBW-Warp@fabric is the highest, reaching 1.63 g/cm^2^/h. The lower water harvesting rates belong to two non-lubricated fabrics, which are BBW-Warp@fabric and Original–Warp@fabric. And Original–Warp@fabric’s water harvesting rate is the lowest (0.76 g/cm^2^/h).

### 3.4. Large Droplet Convergence on 2D and 3D Adjacent Yarns

**Figure 11:**
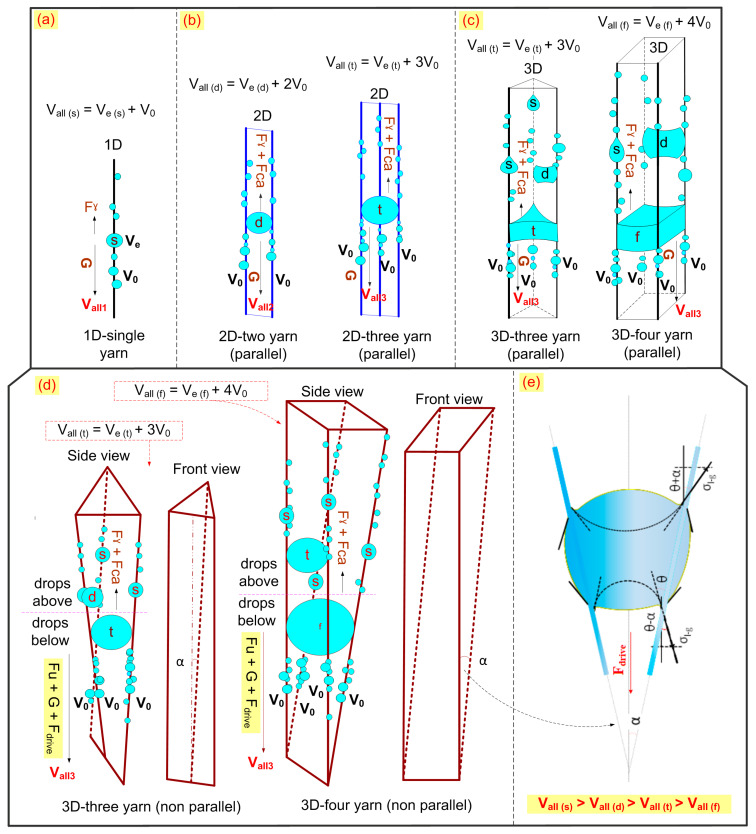
Simulation and illustration of droplet behavior on adjacent filaments and large droplet convergence on adjacent filaments arranged in three dimensions. Adjacent yarns were arranged as follows: (**a**) 1D-single yarn; (**b**) 2D-two yarn (parallel) and 2D-three yarn (parallel); (**c**) 3D-three yarn (parallel) and 3D-four yarn (parallel); (**d**) 3D-three yarn (non-parallel) and 3D-four yarn (non-parallel). And (**e**) Force analysis of water droplets between two adjacent filaments (F_drive_).

**Figure 12:**
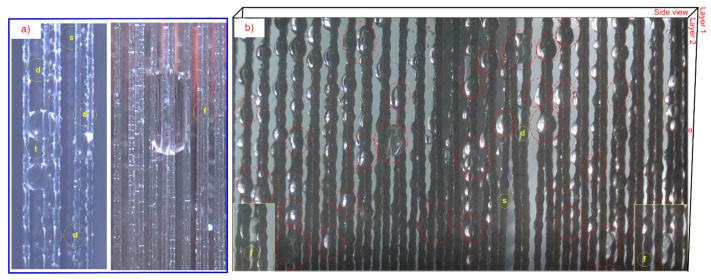
(**a**,**b**) Droplet converging on yarn filament, 2 adjacent yarns, 3 adjacent yarns, and 4 adjacent yarns in 3D on double-layer OBLWB-Warp@fabric and double-layer Vertical Filament Mesh (VFM); angle of inclination between inner and outer fabric layer is α.

Adjacent yarns were arranged as follows: one yarn (1D), two yarns (2D), three yarns (2D), three yarns (3D), and four yarns (3D). Then, the large droplet convergence process and droplet behavior on types of yarns were compared. The tests were conducted on two types of fibers: TPU-filament (Thermoplastic Polyurethane filament) and OBWB yarn. The result of large droplet convergence and behavior was recorded by a camera (Figure 11 and Figure 12):

In our previous work, [25] we demonstrated the advantage of the large droplet converging on two and three adjacent filaments in 2D planar space. Harvested water volume on two and three adjacent filaments in 2D was much larger than that of the droplet on the single filament. The total volume of water shedding down on one, two, and three adjacent yarns is *V_all(s_*_)_ *= V_e(s_*_)_ *+ V_0_*; *V_all(d_*_)_ *= Ve_(d_*_)_ *+ 2V*_0_; and *V_all(t_*_)_ *= V_e(t_*_)_ *+ 3V*_0_: *V_all(t_*_)_ *> V_all(d_*_)_ *> V_all(s_*_)_, respectively (Figure 11). Research by Yutaka Yamada et al. [36] also revealed the role of two and three adjacent yarns in 2D and 3D space in affecting droplet jumping and large droplet convergence. However, their study only considered the case of parallel fibers; non-parallel fibers were not mentioned. For large droplets converging on adjacent yarns in both a 2D plane and 3D space, its existing status (long or short clogging time, shedding down sooner or later) depends on the different forces. These forces consist of gravity (*G*) pulling the droplet down and gravity against forces (capillary forces: *F_ca_* and *F_γ_*). Large drops that converge on many yarns have the advantage because the total amount of water harvested is very large. However, it also has the disadvantage of generating a larger capillary force *F_ca_* and *F_γ_* of a single fiber, causing the droplet to clog for a certain time. According to Furmidge’s theory, the force *F_γ_* is represented as follows [24,48]:

(9) $$\rho_{water}gV_{t}=2\pi R_{f}\gamma (\cos\theta_{r}-\cos\theta_{a})$$

(10) $$\rho_{water}gV_{t}=3\pi R_{f}\gamma (\cos\theta_{r}-\cos\theta_{a})$$

Meanwhile, *ρ_water_* is the density of water, *g* is the gravitational acceleration, *V_t_* is the theoretically obtained critical sliding volume, *R_y_* is the radius of the filament, *γ* is the surface tension of the liquid, *θ_a_* is the forward contact angle, and *θ_r_* is the backward contact angle.

The problem of large droplet clogging on adjacent yarns was solved with two solutions. The highly hydrophobic yarn was lubricated; simultaneously, a three-dimension adjacent yarn structure was arranged by two non-parallel fabric layers. The yarns of the inner and outer layers were intersected at an angle decreasing to zero. Hence, it is possible for the large converged droplet to shed down easily with the help of total force support (*G* + *F_drive_* + *F_u_* (force supported by a slippery surface)), where *F_drive_* is a force analysis of water droplets between two adjacent yarns, expressed through Equation (10) [38]:

(11) $$F_{drive}=2\sigma_{l-g}l\left[\cos(\theta -\alpha /2)-\cos(\theta +\alpha /2\right]$$

where *σ_l_*_-*g*_ is the liquid–gas interfacial tension of a water drop, *α* is the half angle between the two adjacent yarns, *θ* is the contact angle of a water drop on the yarn, and l is the length of the contact line of a water drop inside the yarns. As a result, adjacent yarns in a non-parallel 3D space enabled the droplet to shed down with larger volume, more easily and earlier than the droplets of the parallel and unlubricated adjacent yarns. Furthermore, the non-parallel adjacent yarns have angles decreasing to zero. This also means that the droplets on the upper part of the yarns (droplets above: the distance between yarns is *p* > 2D) will be completely independent of each other. So, initially, the critical droplet volume of single yarn(s) easily sheds downwards to converge into a large droplet (droplets below) (Figure 11c). Moreover, when arranging adjacent yarns in the form of 2D and 3D space, droplets can jump from one yarn to another flexibly, contributing to large droplet convergence. This is consistent with Yutaka Yamada’s research [36]. 

Therefore, the droplet behavior on the 3D fiber system for fog harvesting is optimal, and creates efficient droplet capture, flexible droplet jumping, fast droplet sliding, and droplet converging with a very large volume. In particular, large droplets that converge on three and four yarns (3D) appear quite a lot. This brought the expected results: the water harvesting rate of the OBLWB-Warp@fabric double-layer sample was approximately 27% higher than that of the single-layer OBLWB-Warp@fabric, reaching 2.09 g/cm^2^/h. Also, it was higher than that of the parallel double-layer warp sample (*p* = 2D), 1.82 g/cm^2^/h (in this case, it was only about 11% higher than the single-layer sample). The harvesting rate of double-layer OBLWB-Warp@fabric was 700% higher than that of single-layer Original@fabric. This rate of the double-layer OBLWB-Warp@fabric was about 160% higher than that of Original–Warp@fabric (Figure 10).

## 4. Conclusions

In summary, for the post-weave yarn (wave-bump yarn) of plain weave woven fabric, wetting and surface properties have been modified with tetrabutyl titanate (Ti(OC_4_H_9_)_4_) and PDMS oil. This not only gives the yarn superior droplet development, but also droplet efficiency. This results in OBLWB-Warp@fabric’s fog harvesting rate of 1.63 g/cm^2^/h, about 120% more than Original–Warp@fabric. In addition, a three-dimensional adjacent yarn structure was arranged by two non-parallel fabric layers. The yarns of the inner and outer layers were intersected at an angle decreasing to zero. This helps the droplet behavior on the 3D fiber system for fog harvesting become more optimal, efficient droplet capture, flexible droplet jumping, fast droplet sliding, and droplet converging with a very large volume. This brought the expected results: the water harvesting rate of the OBLWB-Warp@fabric double-layer sample was about 27% higher than that of the single-layer OBLWB-Warp@fabric, reaching 2.09 g/cm^2^/h. Also, it was higher than that of the parallel double-layer warp sample (*p* = 2D), 1.82 g/cm^2^/h (in this case, it was only about 11% higher than the single-layer sample). The harvesting rate of double-layer OBLWB-Warp@fabric was 700% higher than that of single-layer Original@fabric. This rate of the double-layer OBLWB-Warp@fabric was about 160% higher than that of Original–Warp@fabric (Figure 8). This study has revealed the great potential of the post-woven yarn structure; multilayer warp yarn woven fabrics can improve fog harvesting efficiency, with a low cost and ease of installation in practice.

## Data Availability

Data are contained within the article.

## References

[1] Chen Z., Zhang Z. (2020). Recent progress in beetle-inspired superhydrophilic-superhydrophobic micropatterned water-collection materials. Water Sci. Technol. A J. Int. Assoc. Water Pollut. Res..

[2] Katiyar N.K., Goel G., Hawi S., Goel S. (2021). Nature-inspired materials: Emerging trends and prospects. NPG Asia Mater..

[3] Sharma V., Ali-Loytty H., Koivikko A., Yiannacou K., Lahtonen K., Sariola V. (2021). Copper Oxide Microtufts on Natural Fractals for Efficient Water Harvesting. Langmuir ACS J. Surf. Colloids..

[4] Korkmaz S., Kariper İ.A. (2019). Fog harvesting against water shortage. Environ. Chem. Lett..

[5] Riffat S., Powell R., Jarimi H. (2020). Review of sustainable methods for atmospheric water harvesting. Int. J. Low-Carbon Technol..

[6] Hunter P. (2014). Turning nature’s inspiration into a production line. EMBO Rep..

[7] Guo Z., Lei J. (2020). A fog-collecting surface mimicking the Namib beetle: Water collection efficiency and its influencing factors. Nanoscale.

[8] Mitchell D., Henschel J.R., Hetem R.S., Wassenaar T.D., Strauss W.M., Hanrahan S.A., Seely M.K. (2020). Fog and fauna of the Namib Desert: Past and future. Ecosphere.

[9] Park J.K., Kim S. (2019). Three-Dimensionally Structured Flexible Fog Harvesting Surfaces Inspired by Namib Desert Beetles. Micromachines.

[10] Birajdar M.S., Lee J. (2015). Nanoscale Bumps and Dents on Nanofibers Enabling Sonication-Responsive Wetting and Improved Moisture Collection. Macromol. Mater. Eng..

[11] Zhang L., Wu J., Hedhili M.N., Yang X., Wang P. (2015). Inkjet printing for direct micropatterning of a superhydrophobic surface: Toward biomimetic fog harvesting surfaces. J. Mater. Chem. A.

[12] Li J., Zhou Y., Wang W., Du F., Ren L. (2020). A bio-inspired superhydrophobic surface for fog collection and directional water transport. J. Alloys Compd..

[13] Zhu P., Chen R., Zhou C., Tian Y., Wang L. (2021). Asymmetric fibers for efficient fog harvesting. Chem. Eng. J..

[14] Wang J., Yi S., Yang Z., Chen Y., Jiang L., Wong C.P. (2020). Laser Direct Structuring of Bioinspired Spine with Backward Microbarbs and Hierarchical Microchannels for Ultrafast Water Transport and Efficient Fog Harvesting. ACS Appl. Mater. Interfaces.

[15] Klemm O., Schemenauer R.S., Lummerich A., Cereceda P., Marzol V., Corell D., van Heerden J., Reinhard D., Gherezghiher T., Olivier J. (2012). Fog as a Fresh-Water Resource: Overview and Perspectives. AMBIO.

[16] Schunk C., Trautwein P., Hruschka H., Frost E., Dodson L., Derhem A., Bargach J., Menzel A. (2018). Testing Water Yield, Efficiency of Different Meshes and Water Quality with a Novel Fog Collector for High Wind Speeds. Aerosol Air Qual. Res..

[17] Ismail Z., Go Y.I. (2021). Fog-to-Water for Water Scarcity in Climate-Change Hazards Hotspots: Pilot Study in Southeast Asia. Glob. Chall..

[18] Gao Y., Wang J., Xia W., Mou X., Cai Z. (2018). Reusable Hydrophilic-superhydrophobic Patterned Weft Backed Woven Fabric for High-efficiency Water-harvesting Application. ACS Sustain. Chem. Eng..

[19] Yu Z., Zhang H., Huang J., Li S., Zhang S., Cheng Y., Mao J., Dong X., Gao S., Wang S. (2021). Namib desert beetle inspired special patterned fabric with programmable and gradient wettability for efficient fog harvesting. J. Mater. Sci. Technol..

[20] Zhu R., Liu M., Hou Y., Zhang L., Li M., Wang D., Wang D., Fu S. (2020). Biomimetic Fabrication of Janus Fabric with Asymmetric Wettability for Water Purification and Hydrophobic/Hydrophilic Patterned Surfaces for Fog Harvesting. ACS Appl. Mater. Interfaces.

[21] Rivera J.D.D. (2011). Aerodynamic collection efficiency of fog water collectors. Atmos. Res..

[22] Park K.C., Chhatre S.S., Srinivasan S., Cohen R.E., McKinley G.H. (2013). Optimal design of permeable fiber network structures for fog harvesting. Langmuir ACS J. Surf. Colloids.

[23] Ghosh R., Ray T.K., Ganguly R. (2015). Cooling tower fog harvesting in power plants—A pilot study. Energy.

[24] Shi W., Anderson M.J., Tulkoff J.B., Kennedy B.S., Boreyko J.B. (2018). Fog Harvesting with Harps. ACS Appl. Mater. Interfaces.

[25] Nguyen L.T., Bai Z., Zhu J., Gao C., Liu X., Wagaye B.T., Li J., Zhang B., Guo J. (2021). Three-Dimensional Multilayer Vertical Filament Meshes for Enhancing Efficiency in Fog Water Harvesting. ACS Omega.

[26] Nguyen L.T., Bai Z., Zhu J., Gao C., Luu H., Zhang B., Guo J. (2022). Enhancing Fog Harvest Efficiency by 3D Filament Tree and Elastic Space Fabric. ACS Sustain. Chem. Eng..

[27] Nguyen L.T., Bai Z., Zhu J., Gao C., Zhang B., Guo J. (2022). Elastic Textile Threads for Fog Harvesting. Langmuir.

[28] Zhang H., Zhu H. (2012). Modification of wool fabric treated with tetrabutyl titanate by hydrothermal method. J. Text. Inst..

[29] Xue C.H., Jia S.T., Chen H.Z., Wang M. (2008). Superhydrophobic cotton fabrics prepared by sol-gel coating of TiO_2_ and surface hydrophobization. Sci. Technol. Adv. Mater..

[30] Nguyen T.L., Bai Z.Q., Luu H., Nguyen T.T.N., Le T.H., Nguyen T.N., Nguyen T.X., Duong T.T., Tran T., Bin Z. Promoting Droplet Migration and Convergence on Modified Woven Fabric with Nanofiber Strips. Proceedings of the 3rd National Scientific Conference on Textile, Apparel and Leather Engineering (NSCTEX2022).

[31] Srinivasan S., Chhatre S.S., Guardado J.O., Park K.C., Parker A.R., Rubner M.F., McKinley G.H., Cohen R.E. (2014). Quantification of feather structure, wettability and resistance to liquid penetration. J. R. Soc. Interface.

[32] Liu Y., Chen X., Xin J.H. (2008). Hydrophobic duck feathers and their simulation on textile substrates for water repellent treatment. Bioinspir. Biomim..

[33] Yamamoto M., Nishikawa N., Mayama H., Nonomura Y., Yokojima S., Nakamura S., Uchida K. (2015). Theoretical Explanation of the Lotus Effect: Superhydrophobic Property Changes by Removal of Nanostructures from the Surface of a Lotus Leaf. Langmuir ACS J. Surf. Colloids.

[34] Feng S., Delannoy J., Malod A., Zheng H., Quéré D., Wang Z. (2020). Tip-induced flipping of droplets on Janus pillars: From local reconfiguration to global transport. Sci. Adv..

[35] Wang X., Zeng J., Yu X., Liang C., Zhang Y. (2020). Beetle-like droplet-jumping superamphiphobic coatings for enhancing fog collection of sheet arrays. RSC Adv..

[36] Yamada Y., Sakata E., Isobe K., Horibe A. (2021). Wettability Difference Induced Out-of-Plane Unidirectional Droplet Transport for Efficient Fog Harvesting. ACS Appl. Mater. Interfaces.

[37] Han T., Noh H., Park H.S., Kim M.H. (2018). Effects of wettability on droplet movement in a V-shaped groove. Sci. Rep..

[38] Dong H., Zheng Y., Wang N., Bai H., Wang L., Wu J., Zhao Y., Jiang L. (2016). Highly Efficient Fog Collection Unit by Integrating Artificial Spider Silks. Adv. Mater. Interfaces.

[39] Heng X., Luo C. (2014). Bioinspired plate-based fog collectors. ACS Appl. Mater. Interfaces.

[40] Park J., Lee C., Lee S., Cho H., Moon M.W., Kim S.J. (2021). Clogged water bridges for fog harvesting. Soft Matter.

[41] Aziz T., Fan H., Khan F.U., Haroon M., Cheng L. (2018). Modified silicone oil types, mechanical properties and applications. Polym. Bull..

[42] Bolvardi B., Seyfi J., Hejazi I., Otadi M., Khonakdar H.A., Davachi S.M. (2019). Towards an efficient and durable superhydrophobic mesh coated by PDMS/TiO2 nanocomposites for oil/water separation. Appl. Surf. Sci..

[43] Li C., Yu C., Zhou S., Dong Z., Jiang L. (2020). Liquid harvesting and transport on multiscaled curvatures. Proc. Natl. Acad. Sci. USA.

[44] Madaeni S.S., Badieh M.M.S., Vatanpour V., Ghaemi N. (2012). Effect of titanium dioxide nanoparticles on polydimethylsiloxane/polyethersulfone composite membranes for gas separation. Polym. Eng. Sci..

[45] Wang Y., Huang Z., Gurney R.S., Liu D. (2019). Superhydrophobic and photocatalytic PDMS/TiO2 coatings with environmental stability and multifunctionality. Colloids Surf. A Physicochem. Eng. Asp..

[46] Seo D., Lee J., Lee C., Nam Y. (2016). The effects of surface wettability on the fog and dew moisture harvesting performance on tubular surfaces. Sci. Rep..

[47] Cassie A.B.D., Baxter S. (1944). Wettability of Porous Surfaces. Trans. Faraday Soc..

[48] Furmidge C. (1962). Studies at phase interfaces. I. The sliding of liquid drops on solid surfaces and a theory for spray retention. J. Colloid Sci..

